# Myocardial Gene Expression Profiling to Predict and Identify Cardiac Allograft Acute Cellular Rejection: The GET-Study

**DOI:** 10.1371/journal.pone.0167213

**Published:** 2016-11-29

**Authors:** Diane Bodez, Hakim Hocini, Nicolas Tchitchek, Pascaline Tisserand, Nicole Benhaiem, Caroline Barau, Mounira Kharoubi, Aziz Guellich, Soulef Guendouz, Costin Radu, Jean-Paul Couetil, Bijan Ghaleh, Jean-Luc Dubois-Randé, Emmanuel Teiger, Luc Hittinger, Yves Levy, Thibaud Damy

**Affiliations:** 1 AP-HP, Department of Cardiology, Henri Mondor Teaching Hospital, Créteil, France; 2 School of Medicine, Paris-Est-Créteil University (UPEC), Créteil, France; 3 IMRB INSERM U955, Paris-Est-Créteil University (UPEC), Créteil F-94000, France`; 4 DHU ATVB, Henri Mondor Teaching Hospital, Creteil, France; 5 Vaccine Research Institute (VRI), Henri Mondor Teaching Hospital, Créteil, France; 6 CEA, DSV/iMETI, Immunology of viral infections and autoimmune diseases research unit, Fontenay-aux-Roses, France; 7 UMR1184, IDMIT infrastructure, Fontenay-aux-Roses, France; 8 AP-HP, Department of Pathology, Henri Mondor Teaching Hospital, Créteil, France; 9 AP-HP, Platform of Biological Resources, Henri Mondor Teaching Hospital, Créteil, France; 10 GRC Amyloid Research Institute, Henri Mondor Teaching Hospital, Créteil, France; 11 AP-HP, Department of Cardiac Surgery, Henri Mondor Teaching Hospital, Créteil, France; 12 AP-HP, Clinical Immunology, Henri Mondor Teaching Hospital, Créteil, France; Northwestern University, UNITED STATES

## Abstract

**Aims:**

Serial invasive endomyocardial biopsies (EMB) remain the gold standard for acute cellular rejection (ACR) diagnosis. However histological grading has several limitations. We aimed to explore the value of myocardial Gene Expression Profiling (GEP) for diagnosing and identifying predictive biomarkers of ACR.

**Methods:**

A case-control study nested within a retrospective heart transplant patients cohort included 126 patients with median (IQR) age 50 (41–57) years and 111 (88%) males. Among 1157 EMB performed, 467 were eligible (i.e, corresponding to either ISHLT grade 0 or ≥3A), among which 36 were selected for GEP according to the grading: 0 (C_ISHLT_, n = 13); rejection ≥3A (R_ISHLT_, n = 13); 0 one month before ACR (BR_ISHLT_, n = 10).

**Results:**

We found 294 genes differentially expressed between C_ISHLT_ and R_ISHLT_, mainly involved in immune activation, and inflammation. Hierarchical clustering showed a clear segregation of C_ISHLT_ and R_ISHLT_ groups and heterogeneity of GEP within R_ISHLT_. All EMB presented immune activation, but some R_ISHLT_ EMB were strongly subject to inflammation, whereas others, closer to C_ISHLT_, were characterized by structural modifications with lower inflammation level. We identified 15 probes significantly different between BR_ISHLT_ and C_ISHLT_, including the gene of the muscular protein TTN. This result suggests that structural alterations precede inflammation in ACR. Linear Discriminant Analysis based on these 15 probes was able to identify the histological status of every 36 samples.

**Conclusion:**

Myocardial GEP is a helpful method to accurately diagnose ACR, and predicts rejection one month before its histological occurrence. These results should be considered in cardiac allograft recipients’ care.

## Introduction

Cardiac transplantation is the ultimate therapy of end-stage heart failure. Notable advances in immunosuppression allowed significant reduction of both incident and treated acute rejections. However, these therapies are not devoid of severe drawbacks[1] while the incidence of graft rejection remains higher than 25% during the first year post-transplantation with an increased morbidity and mortality.[2] Graft failure and allograft vasculopathy that are related to immune injury, are also main causes of death. Thus, management of allograft rejection is a major clinical concern in the care of heart transplant recipients. Serial endomyocardial biopsies (EMB) for histological examination remain the gold-standard in diagnosing and monitoring acute rejection, based on the 1990 International Society of Heart and Lung Transplantation (ISHLT) classification, revised in 2004.[3] EMB is an invasive procedure, with potential risk of serious complications such as right ventricular perforation or tricuspid regurgitation, and high cost. Moreover, this histopathological assessment is subject to sampling errors and inter-observer variability, especially for EMB with severe rejection. Thus, the ISHLT grading might be not optimal for clinical decisions.[4] Hence, alternative methods are needed for recipients’ management. A number of approaches have been already outlined with a critical focus on using DNA microarray to characterize the gene expression profile (GEP) associated with the ACR.[5–9] Nevertheless, none of these approaches have demonstrated sufficient reliability and feasibility in clinical practice to fully supersede the EMB, whereas interest has been described for risk stratification in heart transplantation[10] or for differential diagnosis improvement in myocardial diseases.[11] In the current study, we investigated GEP of EMB, to find out a genomic signature that could be used for characterizing ACR and predicting its occurrence one month before the histopathological diagnosis.

## Methods

### Patient population

The retrospective cohort of cardiac allograft recipients at Henri Mondor teaching hospital (Créteil, France) between 2003 and 2012 included 126 patients. They received immunosuppressive induction with polyclonal anti-lymphocyte globulin or interleukine-2 receptor antagonist (basiliximab), and the maintenance immunosuppression usually associated prednisone, a calcineurin inhibitor and a cell cycle inhibitor. In some cases, an mTOR inhibitor was used, instead or in addition to calcineurin- or cell cycle inhibitor, or alone with prednisone. Episodes of ACR ≥ grade 2 (ISHLT 1990) were treated with intravenous pulse steroids, change in oral maintenance therapy if needed, and anti-lymphocytes agents or intravenous immunoglobulin in specific cases.

### Sample collection

For routine ACR surveillance, EMB were systematically performed during the follow-up period: monthly the first and quarterly the second year post-transplantation (except in the case of contra-indication, with extra EMB in case of rejection suspicion). Among the 126 patients that underwent cardiac allograft during this period, 21 did not undergo any biopsy because of hemodynamic instability and early death. Among the 105 remaining patients, a total of 1157 biopsies were performed. For each biopsy, 2 to 4 samples were obtained from the right ventricle for ACR histological grading by an experienced cardiac pathologist (NL), according to the 1990 ISHLT criteria since ISHLT guidelines were revised in 2004 during the study period.

### Study design

**Fig 1:**
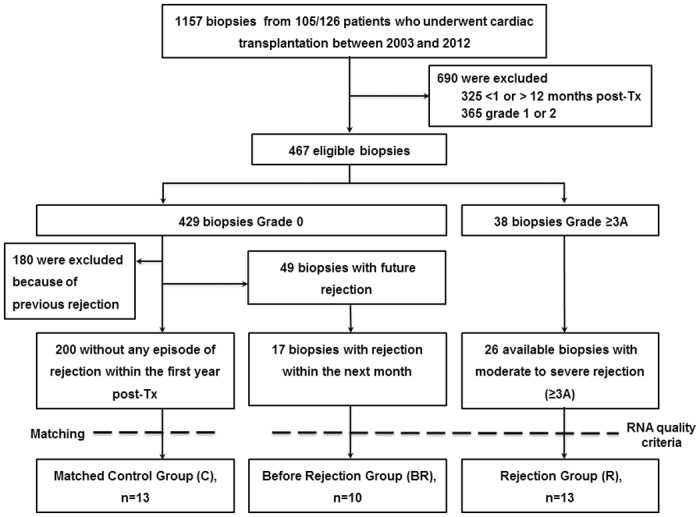
Flow chart of EMB collection from heart transplantation to inclusion in the GET-study.

We conducted a nested case-control study of intragraft gene expression profiles within our cohort of 105 biopsied patients (Fig 1). Study groups were defined as followed: control group (C_ISHLT_): grade 0; rejection group (R_ISHLT_): grade ≥ 3A (i.e. ≥ 2R ISHLT 2004); before rejection group (BR_ISHLT_): grade 0 biopsies followed by a grade ≥ 2 rejection on the next monthly biopsy. To minimize confounders, control samples were obtained from patients without any episode of rejection in the first year following transplantation. For homogeneity reasons, EMB from 1 to 12 months post-transplantation were screened for inclusion. All R_ISHLT_ and BR_ISHLT_ samples of sufficient RNA quality for transcriptome analysis were included (n = 13 and n = 10, respectively). Biopsies of C_ISHLT_ group (n = 13) were matched to other biopsies’ groups for donor’s and recipient’s gender and age, primary cardiac diagnosis, date of transplantation, and time of biopsy from transplantation. Immunosuppressive treatment and all rejection episodes were recorded. The study complied with the Declaration of Helsinki and was approved by the local ethic committee (CPP Ile de France VI). All survival subjects for whom biopsies were used in this study provided their informed written consent. None of the transplant donors were from a vulnerable population.

### RNA isolation and microarray sample preparation

Frozen EMB were immediately immersed into RLT-beta-mercaptoethanol (βME, 1/100) lysis buffer (Qiagen, Courtaboeuf Cedex, France). Samples were then disrupted and homogenized using a TissueLyser LT (Qiagen, Courtaboeuf Cedex, France). RNA was purified with Qiagen RNeasy Micro Kit and quantified using a ND-8000 spectrophotometer (NanoDrop Technologies, Fisher Scientific, Illkirch Cedex, France) before being checked for integrity on a 2100 BioAnalyzer (Agilent Technologies, Massy Cedex, France). cDNA was synthesized and biotin-labeled cRNA was generated by an in vitro transcription reaction using Ambion Illumina TotalPrep RNA Amplification Kits (Applied Biosystem/Ambion, Saint-Aubin, France). Labeled cRNA were hybridized on Illumina Human HT-12V4 BeadChips that targets 47323 probes corresponding to 34694 genes. All steps were done following the manufacturers’ protocols. Microarray row data are available on the EBI-ArrayExpress database under the accession number E-MTAB-5136.

### Statistical analysis

Continuous variables are described as median (interquartile range) and categorical variables in percent (%). Comparisons between groups were made using the Mann-Whitney or the Kruskal-Wallis test for continuous data, and the χ^2^ test for categorical variables. Statistical analyses were done with the use of Prism 6.05 Software (GraphPad Software Inc, La Jolla, CA). P-values ≤0.05 were considered significant. The inter-observation variability for histological analysis was assessed by calculating the weighted kappa coefficient between two blind readings. Microarray data analyses were performed blind to clinical data using R software version 3.0.1. Raw data were quantile normalized via the Limma package:[12] first background subtraction, then log2 transformation and quantile normalization were applied. A gene was considered as differentially expressed 1) when a comparison between two groups yielded a P-value ≤ 0.05 in the student parametric test and 2) a variation of at least 1.3-fold was observed. Fold-change was raised to 1.5 for the comparison between Rejection and Control groups in order to restrict the number of probes differentially expressed. Hierarchical clustering was performed on scaled gene expression based on the Euclidean distance and the Ward’s linkage method. Canonical pathways and biological functions associated with differentially expressed genes were identified using Ingenuity Pathway Analysis software (IPA^®^, Qiagen, Redwood City, www.qiagen.com/ingenuity). Linear Discriminant Analysis (LDA) was further performed using the ‘MASS’ package with the intention of predicting EMB histological features from their individual gene expression levels.

## Results

### Patient characteristics

According to inclusion criteria, 467 EMB were eligible (i.e. corresponding to either ACR grade 0 or ≥ 3A between 1 and 12 months post-transplantation). Among them (Fig 1), 429 showed no evidence of ACR (grade 0) and 38 showed rejection ≥ grade 3A. In R_ISHLT_ and BR_ISHLT_ groups, all samples reaching quality criteria for microarray analysis were included. Samples of the C_ISHLT_ group were matched to those previously included in the other groups. Thirty-six biopsies belonging to 30 patients were studied (group C_ISHLT_, n = 13; group R_ISHLT_, n = 13; group BR_ISHLT_, n = 10). Weighted kappa coefficient evaluating the agreement between two histological readings of these biopsies was 0.828. The 30 patients included for GEP analysis were not different from the 75 biopsied patients without GEP (Table 1). Among the 30 patients, 22 were male (73.3%). Age at the time of EMB was 52.1(34.5;57.2) years. Time between transplantation and EMB was 4.1(1.5;5.8) months and median EMB conservation time before RNA purification was 52.4(21.4;79.2) months. Samples of the BR_ISHLT_ group were collected 34(29;35) days before the episode of rejection. RIN (RNA Integrity Number) was 6.2(5.8;6.5). Characteristics of the 36 biopsies and relative patients are presented in Table 2. Comparisons of 3 groups’ characteristics are given in Table 3. There were no significant differences between the 3 groups with the exception of proportion of mycophenolate mofetil-treated patients.

**Table 1:** Baseline characteristics of the patients with EMB according to selection for GEP analysis.

Variables/Heart Transplant patients	Overall EMB patients	EMB patients without GEP	EMB patients with GEP	*P*-value
*n*	105	75	30	
Recipient age, years	50 (39;57)	50 (40;57)	50 (40;56)	.74
Recipient sex, male, n(%)	84 (80)	61 (81)	22 (73)	.60
Primary cardiac diagnosis				.50
• Idiopathic dilated cardiomyopathy	41 (39)	25 (33)	16 (53)	
• Ischemic cardiomyopathy	36 (34)	28 (37)	8 (27)	
• Others	28 (27)	22 (29)	6 (20)	

EMB: endomyocardial biopsy; GEP: gene expression profiling

**Table 2:** Baseline characteristics of the patients with EMB selected for GEP analysis.

Sample	ISHLT (1990)	Patient age (y)	Donor age (y)	Recipient gender	Donor gender	Transplant status	Time from Tx (months)	Prednisone (mg/d)	Cyclosporine (mg/d)	MMF (g/d)	Tacrolimus (mg/d)	Everolimus (mg/d)	Sirolimus (mg/d)	Induction
1	0	23	33	F	F	BR	5.1	8	250	-	-	-	-	ATG
2	0	31	16	M	M	C	2.2	25	475	2.5	-	-	-	ATG
3	0	50	54	M	M	C	8.1	5	-	1.5	3	-	-	ATG
4	0	31	16	M	M	C	5.6	10	425	1.5	-	-	-	ATG
5	0	32	43	M	F	BR	5.0	20	350	2.0	-	-	-	ATG
6	3A	53	31	M	M	R	1.4	25	275	2.25	-	-	-	ATG
7	3A	58	16	M	M	R	4.6	25	-	3.0	5	-	-	ATG
8	3A	33	43	F	F	R	1.3	30	300	3.0	-	-	-	ATG
9	0	59	21	M	M	BR	1.0	30	-	-	1	-	-	ATG
10	3A	50	20	M	F	R	1.5	20	300	1.0	-	-	-	ATG
11	3A	59	21	M	M	R	4.2	10	-	1.0	6	1.5	-	ATG
12	3A	42	59	M	F	R	6.9	5	-	1.0	-	-	1	ATG
13	0	64	60	M	F	BR	10.9	10	-	-	7	-	-	ATG
14	3A	64	60	M	F	R	11.7	5	-	-	7	-	-	ATG
15	3A	34	58	M	F	R	1.0	25	-	2.0	4	-	-	IL2R-I
16	0	53	25	M	M	C	3.9	20	-	1.5	5	-	-	IL2R-I
17	0	57	56	M	F	C	2.0	15	-	2.0	8	-	-	IL2R-I
18	0	61	61	F	F	BR	8.2	15	-	1.5	4	-	-	IL2R-I
19	3A	54	33	M	M	R	1.1	50	-	1.0	2	-	-	IL2R-I
20	3A	34	58	M	F	R	5.3	15	-	0.72	2	-	-	IL2R-I
21	0	57	62	M	M	C	2.1	20	-	3.0	2	-	-	IL2R-I
22	0	54	33	M	M	BR	6.0	20	-	-	5	2	-	IL2R-I
23	0	53	53	M	M	BR	5.5	15	250	3.0	-	-	-	ATG
24	0	35	42	M	M	C	2.1	35	-	2.0	5	-	-	ATG
25	0	24	50	F	F	C	3.4	25	150	1.0	-	-	-	ATG
26	0	24	50	F	F	C	6.2	15	150	1.0	-	-	-	ATG
27	3A	44	21	M	M	R	5.1	45	375	3.0	-	-	-	IL2R-I
28	3A	51	53	M	F	R	7.7	10	-	3.0	-	1	-	ATG
29	0	49	20	F	F	BR	1.4	25	200	2.5	-	-	-	ATG
30	0	39	51	M	M	BR	0.2	25	-	1.0	6	-	-	IL2R-I
31	0	51	17	F	M	C	1.3	65	350	2.0	-	-	-	ATG
32	0	59	46	F	F	BR	1.2	40	150	1.5	-	-	-	ATG
33	0	57	18	M	M	C	2.7	25	-	1.0	8	-	-	ATG
34	3A	57	62	F	F	R	8.2	10	150	0.5	-	-	-	ATG
35	0	61	52	M	F	C	4.6	15	-	2.0	16	4	-	ATG
36	0	57	60	M	F	C	3.7	15	-	1.5	5	-	-	ATG

ATG: Anti-thymoglobulin; BR: before rejection (BR _ISHLT_); C Control (C_ISHLT_); EMB: endomyocardial biopsy; GEP: gene expression profile; F: female; ISHLT: International Society of Heart and Lung Transplantation; M: Male; MMF: mycophenolate mofetil; IL2R-I: IL2 receptor inhibitor; R: rejection (R _ISHLT_); Tx: transplantation

**Table 3:** Baseline characteristics of the EMB having GEP determine according to histological status.

Variables/Groups	C_ISHLT_	R_ISHLT_	BR_ISHLT_	*P*-value
*n*	13	13	10	
Recipient age, years	52 (31;57)	51 (38;58)	54 (37;60)	0.61
Recipient sex, male, n(%)*	9 (82)	10 (83)	7 (70)	0.72
Donor sex, male, n(%)*	7 (64)	5 (42)	4 (40)	0.47
EMB conservation time, months	25.0 (8.2;73.5)	65.9 (36.6;80.1)	48.3 (24.9;83.0)	0.48
Time from transplantation, months	3.4 (2.1;5.1)	4.6 (1.4;7.3)	5.1 (1.2;6.6)	0.93
Immunosuppressive therapy at EMB time				
• Prednisone, n(%)	13 (100.0)	13 (100.0)	10 (100.0)	1.00
• Calcineurin inhibitor, n(%)	13 (100.0)	11 (84.6)	10 (100.0)	0.15
• Mycophenolate mofetil, n(%)	13 (100.0)	12 (92.3)	6 (60.0)	0.02
• mTOR inhibitor, n(%)	1 (7.7)	3 (23.1)	1 (10.0)	0.48
Induction therapy, any, n(%)*	11 (100)	12 (100)	10 (100)	1.00
• Anti-thymoglobulin, n(%) (vs Basiliximab)	8 (72.7)	9 (75.0)	7 (70.0)	0.97

* Based on number of patient per group. ISHLT: International Society of Heart and Lung Transplantation; EMB: endomyocardial biopsy; other abbreviations as in Table 2

### Distinctive gene expression profiles between rejection and control groups

**Fig 2:**
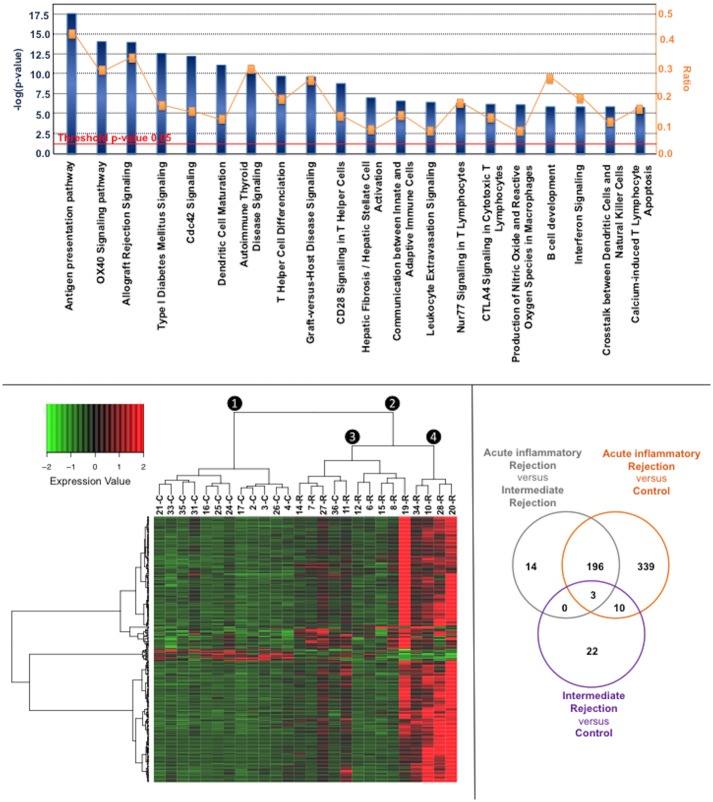
Comparison of gene expression profiles between C_ISHLT_ and R_ISHLT_ groups. **2A** (top). Top 20 canonical pathways significantly over-represented in R_ISHLT_ compared to C_ISHLT_ group, listed in the order of–log(p-value) of over-representation (the first pathway being the more significantly over-represented). Ratio indicates the number of genes differentially expressed related to the number of genes known to be part of the pathway. **2B** (left bottom). Heatmap with hierarchical clustering for the 341 probes differentially expressed between C_ISHLT_ and R_ISHLT_ samples. Gene expressions row scaled. **2C** (right bottom). Overlaps of differentially expressed genes between the three clusters Control (❶), intermediate Rejection (❸), and acute inflammatory Rejection (❹).

**Fig 3:**
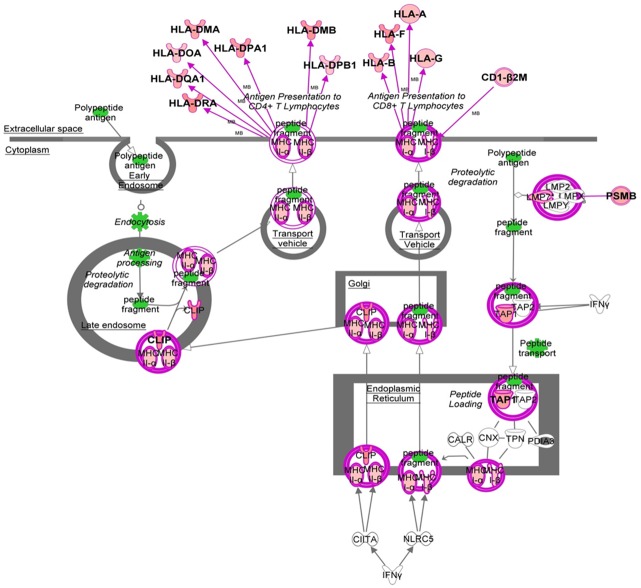
Overview of the IPA Antigen Presentation Pathway. Red color indicates an up-regulation (fold-change ≥1.5), and color intensity is proportional to the fold-change. Shapes: triangles represent kinases; squares represent cytokines; rectangles represent ligand-dependent nuclear receptors; diamonds represent enzymes; trapezoids represent transporters; ellipses represent transcription regulators; and circles represent others molecules. Double outline indicates complex of molecules. Polypeptide antigen is followed in green until antigen presentation. Cellular structures are underlined, the steps of the pathway are in italics. Differentially expressed genes are in bold one time in the pathway.

We found 341 probes corresponding to 294 annotated genes differentially expressed between C_ISHLT_ and R_ISHLT_. Most differentially expressed genes were Chemokines CXCL9 and CCL5, and Lymphotoxin Beta (LTB), all upregulated in 5.3- to 7.3-fold in the R_ISHLT_ group (Table 4). Considering all, genes differentially expressed between C_ISHLT_ and R_ISHLT_ groups basically involved in antigen presentation (HLA-DR, HLA-DM, HLA-DP, TAP1), lymphocytes recruitment (CXCL9, CCL5, CCL8, CD44), T-cell activation (LTB, CD247, CD86) and Interferon responses (CXCL9, STAT1, IRF8, IRF1), as depicted in Fig 2A. The antigen presentation pathway was particularly represented with 15 genes differentially expressed among the 36 genes known to be part of this pathway (Figs 2A and 3).

**Table 4:** Top list of differentially expressed genes between C_ISHLT_ and R_ISHLT_ groups.

	Gene symbol	Gene name	Function	FC
**1**	CXCL9	Chemokine (C-X-C motif) ligand 9	T-cell, chemokine	7.3
**2**	LTB	Lymphotoxin bêta	Lymphoid response	5.6
**3**	CCL5	Chemokine (C-C Motif) Ligand 5 (= RANTES)	T-cell, chemokine	5.3
**4**	CD3D	CD3 Delta Chain (CD3-TCR complex)	T-cell	4.8
**5**	LCP1	Plastin-2	Hematopoietic cell lineages	4.5
**6**	RAC2	Ras-related C3 botulinum toxin substrate 2	Small signalling G protein	4.3
**7**	RARRES3	Retinoic acid receptor responder protein 3	Cellular differentiation	4.3
**8**	CD8A	Cluster of Differentiation 8A	Cytotoxic T-cell	4.1
**9**	STAT1	Signal Transducer And Activator Of Transcription 1	Interferon responses	4.0
**10**	ITGB2	Integrin bêta-2 (CD18)	Cell adhesion, cell-surface signalling	3.6
**11**	HCST	Hematopoietic cell signal transducer	Hematopoietic cell	3.5
**12**	CTSC	Cathepsin C	Serine proteases in immune cells	3.3
**13**	CD52	CAMPATH-1 antigen	Mature lymphocytes, monocytes, dendritic cells	3.3
**14**	CD74	HLA-class II histocompatibility antigen, DR invariant chain	Antigen presentation	3.3
**15**	AIF1	Allograft inflammatory factor 1	IFN gamma responses	3.2
**16**	HLA-DRA	HLA-class II histocompatibility antigen, DR alpha chain	Antigen presentation	3.1
**17**	PLEK	Pleckstrin	Haemostasis	3.1
**18**	GZMA	Granzyme A	Cytotoxicity	3.0
**19**	VCAM1	Vascular cell adhesion protein 1, (= CD106)	Endothelial leucocytes adhesion	3.0
**20**	THY1	Thymocyte antigen 1, (= CD90)	Thymocytes, pro-thymocytes	3.0

FC: Fold-change

We used hierarchical clustering to assess the ability of the 341 probes to distinguish control and rejection samples. As shown in Fig 2B, samples clustered clearly into 2 main clusters (❶, ❷) corresponding to C_ISHLT_ and R_ISHLT_ samples, respectively. One C_ISHLT_ sample clustered with R_ISHLT_ samples; it has been considered according to its gene expression profile close to R_ISHLT_ samples rather than its pathological grading, in the further GEP analyses.

### Heterogeneity of gene expression profiles among rejection samples

Fig 2B shows that samples of C_ISHLT_ and R_ISHLT_ clustered in sub-clusters rather than into single branches, indicating heterogeneity of cardiac gene expression within each group despite an homogeneous histological classification of C_ISHLT_ (grade 0) and R_ISHLT_ (grade 3A). Comparison between the most extreme gene expression profiles, cluster Control ❶ and sub-cluster Rejection ❹, showed that 548 annotated genes were differentially expressed, mainly involved in antigen presentation, immune activation and inflammatory response. Interestingly, the same pathways were associated with the 213 annotated genes differentially expressed between sub-clusters Rejection ❸ and ❹. Besides, only 35 annotated genes differentiated Cluster Control ❶ to sub-cluster Rejection ❸, making this intermediate Rejection cluster (❸) closer to the Control cluster (❶) than to the acute inflammatory Rejection cluster (❹). Fig 2C shows the overlap of genes differentially expressed in the three comparisons. Twenty-two genes were thus identified to be specific of the sub-cluster intermediate Rejection ❸ (Table 5). Two genes were associated to the cardiovascular system (NPPA, NPPB), 3 to immune response (CD3D, CD8A, CD74), and 9 to the extra-cellular matrix (COL3A1, COL1A2, LUM, BGN, COL6A2, FBLN2, ECM1, APOD, BGN).

**Table 5:** List of 22 genes exclusively differentially expressed between Control cluster ❶ and Intermediate Rejection sub-cluster ❸.

	Gene symbol	Gene name	Function	FC
**1**	COL3A1	Collagen alpha-1(III) chain	Connective tissue	2.7
**2**	COL1A2	Collagen alpha-2(I) chain	Connective tissue	2.4
**3**	CD74	HLA-class II histocompatibility antigen, DR invariant chain	Antigen presentation	2.2
**4**	NPPB	Natriuretic peptide B	Neuropeptide hormone	2.1
**5**	NPPA	Natriuretic peptide A	Neuropeptide hormone	2.1
**6**	CD3D	CD3 Delta Chain (CD3-TCR complex)	T-cell	2.1
**7**	CD8A	Cluster of Differentiation 8A	Cytotoxic T-cell	1.8
**8**	LUM	Lumican	Extra-cellular matrix protein	1.7
**9**	BGN	Biglycan	Extra-cellular matrix binding	1.7
**10**	COL6A2	Collagen alpha-2(VI) chain	Connective tissue	1.7
**11**	FBLN2	Fibulin-2	Extra-cellular matrix protein	1.7
**12**	EPHA3	Ephrin type-A receptor 3	Protein-Tyrosine kinase	1.6
**13**	AEBP1	Adipocyte enhancer-binding protein 1 precursor	Smooth muscle cell differentiation	1.6
**14**	SCD	Stearoyl-CoA desaturase (delta-9-desaturase)	Fatty acid metabolism	1.6
**15**	APOBEC3F	DNA dC->dU-editing enzyme APOBEC-3F	Innate immune response	1.6
**16**	ECM1	Extra-cellular matrix protein 1	Extra-cellular matrix protein	1.5
**17**	UHFR1	Ubiquitin-like with PHD and ring finger	Chromatin structure regulation	1.5
**18**	WDR62	WD repeat-containing protein 62	Cerebral cortical development	-1.5
**19**	STXBP6	Syntaxin binding protein (amisyn)	Exocytosis	-1.6
**20**	APOD	Apolipoprotein D	Lipoprotein metabolism	-1.7
**21**	CSHL1	Chorionic somatomammotropin hormone-like 1	Transcription regulator	-1.8
**22**	HMGCS2	3-hydroxy-3-methylglutaryl-CoA synthase 2	Ketogenesis	-2.5

FC: Fold-change

### Gene expression signature one month before rejection

**Fig 4:**
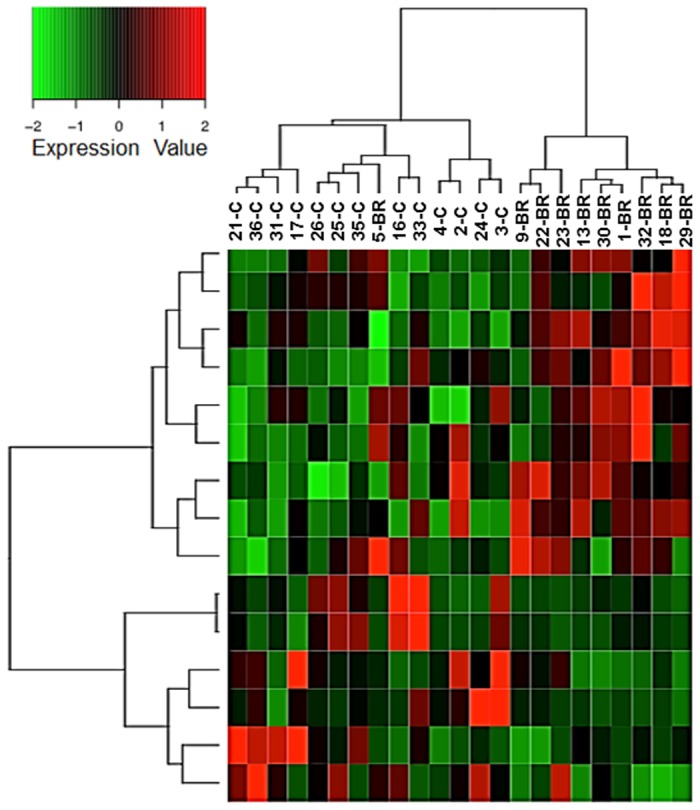
Heatmap of gene expression with hierarchical clustering for the 15 probes differentially expressed between C_ISHLT_ and BR_ISHLT_ samples. Gene expressions row scaled.

Comparison of GEP between C_ISHLT_ and BR_ISHLT_ showed 15 differentially expressed probes, corresponding to 11 annotated genes (Table 6) of which fold-changes absolute values were higher than 1.3. Among them, MAL that is involved in T-cell signal transduction, TTN involved in striated muscle contraction and PI16 that negatively regulate cell growth involved in cardiac muscle cell development and also plays a role in Treg functions. Based on the expression level of these 15 probes, hierarchical analysis was able to segregate samples into two main clusters corresponding to C_ISHLT_ and BR_ISHLT_ groups, respectively (Fig 4).

**Table 6:** List of 11 annotated differentially expressed genes between C_ISHLT_ and BR_ISHLT_ groups.

	Gene symbol	Gene name	Function	FC
**1**	MAL	Myelin and lymphocyte protein	T-cell signal transduction	1.5
**2**	NR1D2	Nuclear Receptor Subfamily 1, Group D, Member 2	Transcription regulator	1.4
**3**	C5ORF13	Neuronal regeneration related protein (= NREP)	Neuronal regeneration	1.4
**4**	TTN	Titin	Striated muscle contraction	1.4
**5**	TBX2	T-box 2	Developmental processes regulation	1.4
**6**	FRMD3	FERM Domain Containing 3	Tumor suppressor	1.3
**7**	F3	Coagulation factor III	Coagulation	1.3
**8**	CDH13	Cadherin Heart 13	Cytoskeleton reorganization	1.3
**9**	SNCA	Alpha-synuclein	Presynaptic signalling	-1.3
**10**	IFI6	Interferon alpha-inducible protein 6	Apoptosis regulator	-1.3
**11**	PI16	Peptidase Inhibitor 16	Memory T regulator	-1.7

FC: Fold-change

### Linear discriminant model to segregate C, BR and R samples

**Fig 5:**
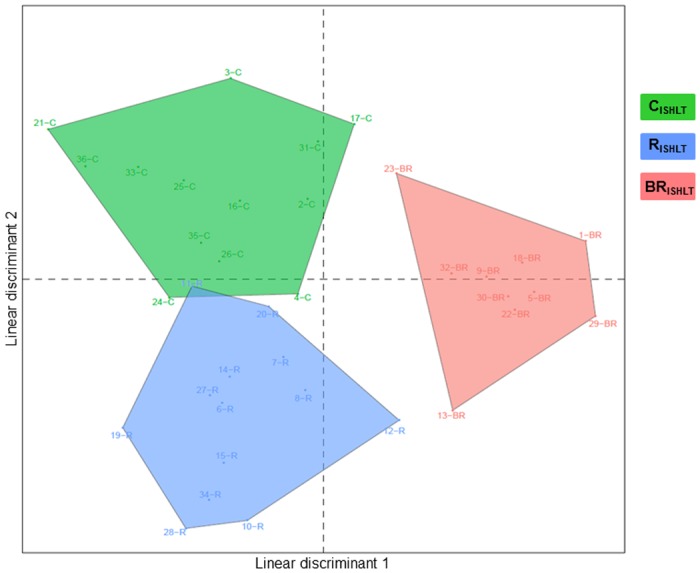
Representation of Linear discriminant analysis of C_ISHLT_, R_ISHLT_ and BR_ISHLT_ samples. Expression profiles were restricted to the set of 15 probes.

In order to estimate the ability of this 15 probes signature to segregate all the conditions, we performed a LDA based on the individual expression levels of 15 probes in each EMB. The analysis was able to discriminate clearly biopsies from each histological group (Fig 5). Moreover, LDA can identify BR_ISHLT_ samples, meaning that a histological rejection episode can be predicted one month before its occurrence.

## Discussion

In the current study we show first that GEP discriminates C_ISHLT_ and R_ISHLT_ EMB. Second, GEP analysis reveals a heterogeneous profile among the histological homogeneous group of R_ISHLT_ EMB, with a possible continuum from intermediate rejection characterized by heart structural modifications to acute inflammatory rejection features. Third, taking the opportunity to explore the GEP of EMB classified as grade 0, in a median of 34 days before rejection, we provide evidence that GEP changes might precede histological features. Moreover, the low number of genes differentially expressed before histological diagnostic of rejection, highlighting structural and immune modulations, allows to define a gene signature predicting ACR. Finally, using LDA, we show that this signature could be sufficient and of higher value for discriminating the three groups of EMB analyzed in this study.

### Molecular ACR signature

The first part of our analysis highlighted a strong molecular immune activation during ACR that is consistent with the previously described pathophysiology of ACR.[13] Compared to other studies that explored gene expression in EMB, we found some identical immune pathways[8] such as the antigen presentation and dendritic cell maturation. Among genes differentially expressed with highest fold changes, were the CXCL9, interferon-gamma-inducible T-cell chemoattractant, and CCL5 (RANTES) that chemo-attracts and induces activation of T-cells and monocytes. Accordingly, these chemokines have been associated with allograft rejection. Urinary CXCL9 protein have been recently reported to have a strong predictive value for non-invasively diagnosing T cell-mediated kidney allograft rejection;[14] CCL5 have been described in cardiac allograft rejection, both in human[15–17] and animal models[18–20] where suppression of CCL5-mediated signals can alleviate transplant rejection severity. Interestingly, maraviroc, an antagonist of the CCL5 receptor CCR5 has been approved for clinical use in the treatment of HIV-infected patients,[21] and is investigated in graft-versus-host disease.[22] Hence, our results support the potential effects of maraviroc in the treatment of cardiac allograft ACR, and provide targets for new therapeutic strategies.

Interestingly, none of the differentially-regulated genes identified in our study overlap with the gene panel used in the Allomap assay, the only test recommended for the non-invasive monitoring of ACR. However the Allomap assay is based on genes differentially expressed in the whole blood, which is probably very different from genes differentially expressed in the myocardium, as it has already been suggested.[6] Results from our study, focusing on myocardial GEP ability to predict and characterize ACR, and the IMAGE Study,[7] focusing on peripheral biomarkers of ACR, are thus not easily comparable.

### Molecular heterogeneity of severe ACR

Interestingly, our results brought out the GEP heterogeneity among the R_ISHLT_ group: one sub-cluster was characterized by a strong immune activation and an interferon-mediated inflammatory state, while another was very close to the control group considering the fewer number of genes differentially expressed. This last sub-cluster was characterized by lower level of inflammation, an incipient cellular response, in addition to extracellular matrix changes that could be of great interest for clinical management. Besides, we found in this cluster an up-regulation of genes of natriuretic peptides ANP and BNP, currently used as biomarkers of heart disease.[23] Meirovich et al have already reported that BNP plasma levels were increased during a cardiac ACR episode grade 3A[24], promoted by pro-inflammatory and other cytokines including CCL5. Regarding the current results, natriuretic peptides up-regulation may be considered as reflect of myocardial stress preceding inflammatory signals during acute rejection. Given a good agreement between the two blind histological readings, heterogeneity in myocardial GEP among the rejection group appears to be unlikely due to miss-reads of the histological findings.

Taken together, these results indicate that GEP characterizes more in-depth molecular pattern of ACR than histopathology. Mengel[25] and Holweg[8] reported a discrepancy between the current ACR ISHLT histological grading system and the myocardial molecular profiles, and also its lack of clinical relevance. This discrepancy and the associated lack of clinical relevance might thus be related to an insufficient sub-grading of ACR with pathological examination, highlighted by our results. Considering adverse effects of immunosuppressive therapies on one hand, and the decrease of the incidence of treated rejection during the past decade,[2] identifying ACR that need reinforced and/or targeted immunosuppressive therapy according to rejection stage is of crucial importance.

### Cardiac molecular signs precede histological ACR alterations

In the second part of our study, we tested the ability of GEP analysis to identify, among samples without any history or current sign of ACR (grade 0), those evolving to rejection one month later. We demonstrated that mRNA expression pattern in EMB is modified one month before the histological rejection features. Among the 11 annotated genes differentially expressed, we found an up-regulation of TTN known to be the defining structural protein of the sarcomere. As a key element of myocardial passive tension, it is involved in the pathogenesis of heart disease, [26,27] and recently recognized as the major human disease gene for dilated cardiomyopathy.[28] We also found up-regulation of several collagen genes in the “intermediate” rejection sub-cluster, suggesting that early structural changes precede inflammatory response. Thus, up-regulation of TTN could be considered as an early marker of myocardial impairment. Further studies are needed to determine whether it is a non-immunological sign of rejection, or a “danger signal” leading to rejection. Besides, we found PI16 down-regulated in the BR_ISHLT_ condition. Interestingly, the relative protein has been reported in cardiac muscle cell development[29] and also associated to functional memory regulatory T cells[30]. We can thus suggest that the under-expression of PI16 reduces its inflammatory regulation properties, and promotes ACR occurrence one month later.

Very few studies explored the potential of GEP in ACR prediction,[31,32] and they all used gene expression profiling score from Allomap test to distinguish patients with future rejection episode from those with persistent histologic quiescence. In contrast to the present study, none was specifically designed to explore, without any a priori notion, the specific molecular pattern of EMB before rejection. Thus, to our knowledge, the present results are the first to report that EMB, one month prior to an episode of ACR, presented a specific molecular pattern that can be used to distinguish them from long-term non-rejecting EMB. Applied to clinical settings, these results might guide actual protocol of EMB surveillance. Predicting immune event one month before might help starting specific treatment at an earlier stage, before occurrence of tissue damages.

### Limitations

We acknowledge several limitations to this study. First the sample size was small, but consistent with what is usually done in GEP studies.[5,6,8] The reason is partly due to limited availability of EMB. Because of the small sample size, we were not able to perform subgroup analyzes and thus we did not assess the influence of confounding factors such as immune-suppressive therapy on changes in intragraft gene expression. However, age, gender, experimentation and sample conservation conditions were proved to behave as random in an ANOVA analysis. Second, we did not focus on antibody-mediated rejection (AMR) because none of the included patients did present AMR in their post-transplantation history, and there we are not able to confront our results to previous studies on endomyocardial biomarkers of AMR [33]. Lastly, gene expression results in our work are compared to the EMB pathological analysis, which is a poor gold standard, especially given the possible focal distribution of histological lesions. Nevertheless we can expect the myocardial GEP distribution to be more homogeneous than histological lesions.

## Conclusion and Perspectives

This study demonstrated that cardiac gene expression profiles matched partly the histological grading system, suggesting earlier and more sensitive performances in diagnosing ACR. Thus, cardiac GEP might provide an early screening test for ACR. Extension of this approach to peripheral blood and other types of rejection may be useful in clinical practice. Further large-scale studies are required to confirm the cardiac molecular signature of ACR in blood for developing a clinically accurate and non-invasive test to predict and diagnose acute cardiac allograft rejection.
